# Bisphosphonate affects the behavioral responses to HCl by disrupting farnesyl diphosphate synthase in mouse taste bud and tongue epithelial cells

**DOI:** 10.1038/s41598-022-25755-5

**Published:** 2022-12-08

**Authors:** Asami Oike, Shusuke Iwata, Ayaka Hirayama, Yurika Ono, Yuki Nagasato, Yuko Kawabata, Shingo Takai, Keisuke Sanematsu, Naohisa Wada, Noriatsu Shigemura

**Affiliations:** 1 Section of Oral Neuroscience, Graduate School of Dental Science, Kyushu University, Fukuoka, Japan; 2 Section of Interdisciplinary Dentistry, Graduate School of Dental Science, Kyushu University, Fukuoka, Japan; 3 Research and Development Center for Five-Sense Devices, Kyushu University, Fukuoka, Japan; 4 Department of Cell Biology, Aging Science, and Pharmacology, Division of Oral Biological Sciences, Faculty of Dental Science, Kyushu University, Fukuoka, Japan; 5 Oral Health/Brain Health/Total Health Research Center, Faculty of Dental Science, Kyushu University, Fukuoka, Japan

**Keywords:** Taste receptors, Perception, Feeding behaviour

## Abstract

Little is known about the molecular mechanisms underlying drug-induced taste disorders, which can cause malnutrition and reduce quality of life. One of taste disorders is known adverse effects of bisphosphonates, which are administered as anti-osteoporotic drugs. Therefore, the present study evaluated the effects of risedronate (a bisphosphonate) on taste bud cells. Expression analyses revealed that farnesyl diphosphate synthase (FDPS, a key enzyme in the mevalonate pathway) was present in a subset of mouse taste bud and tongue epithelial cells, especially type III sour-sensitive taste cells. Other mevalonate pathway-associated molecules were also detected in mouse taste buds. Behavioral analyses revealed that mice administered risedronate exhibited a significantly enhanced aversion to HCl but not for other basic taste solutions, whereas the taste nerve responses were not affected by risedronate. Additionally, the taste buds of mice administered risedronate exhibited significantly lower mRNA expression of *desmoglein-2*, an integral component of desmosomes. Taken together, these findings suggest that risedronate may interact directly with FDPS to inhibit the mevalonate pathway in taste bud and tongue epithelial cells, thereby affecting the expression of desmoglein-2 related with epithelial barrier function, which may lead to alterations in behavioral responses to HCl via somatosensory nerves.

## Introduction

Taste disorders can reduce quality of life and cause loss of appetite, which in turn may lead to a deterioration in nutritional status and a decline in activities of daily living. Drug-induced taste disorders are the most frequent cause of taste disturbances. More than 280 drugs have been reported to cause alterations in human taste perception^1^. Bisphosphonates are widely used to treat osteoporosis but are known to cause transient taste loss or changes in taste perception (metallic taste), and bisphosphonates have been reported to induce dysgeusia in about 5% of patients with osteoporosis^2–4^. In addition, patients with cancer who are prescribed bisphosphonates also show changes in taste and odor perception^5^. However, little is known about the molecular mechanisms underlying bisphosphonate-induced taste disorders.

Bisphosphonates inhibit farnesyl diphosphate synthase (FDPS), which is a key enzyme in the mevalonate pathway that mediates cholesterol synthesis. Inhibition of FDPS reduces the formation of isoprenoid lipids such as farnesyl diphosphate and geranylgeranyl pyrophosphate, which decreases the prenylation of small GTPases such as Rho, Ras and Rac that are essential for various cellular functions including osteoclast migration, proliferation and differentiation; these effects of FDPS lead to osteoclast apoptosis and a reduction in bone resorption^6,7^. Interestingly, recent studies have shown that bisphosphonates also affect epithelial adhesion, differentiation, proliferation, migration, senescence and apoptosis in the oral mucosa of healthy individuals and in cultured human oral keratinocytes^8^.

Taste buds distributed on the epithelium of the tongue and palate each consist of 50–100 taste cells innervated by taste nerves. Recent molecular biological studies have identified candidate receptors and taste-related molecules that are expressed in the taste cells for the five basic tastes^9–11^. Taste receptors are divided into two types: G protein-coupled receptors, which include sweet taste receptors (taste receptor type 1 member 2 [T1R2] and T1R3), bitter taste receptors (type II taste receptors) and umami taste receptors (T1R1 and T1R3); and channel receptors, which include salty taste receptors (epithelial sodium channel [ENaCα]) and sour taste receptors (otopetrin-1 [Otop1]). Taste transduction is considered to occur at the level of the taste cell through activation of each taste receptor and taste-related molecules^10^. Comprehensive expression analyses using RNA-sequencing have revealed the genes expressed in each type of mouse taste cell^12^, which showed that FDPS and other mevalonate pathway genes [*3-hydroxy-3-methylglutaryl-CoA reductase* (*HMGCR*), *mevalonate kinase* (*Mvk*) *and squalene synthase* (*FDFT*)] are highly expressed in both T1R3 positive type II and sour sensitive type III taste cells at least at the level of mRNA^12^.

We thus hypothesized that the FDPS and other mevalonate pathway genes are involved in the mechanisms underlying the adverse effects of bisphosphonates on the taste system^12^. We explored the role of FDPS function in mouse taste buds by examining the effects of administering risedronate (a representative bisphosphonate) for 28 days on the number of taste bud cells, the behavioral and neural responses of mice to taste stimuli, and the expression levels of taste cell markers and intercellular adhesion molecules (desmogleins).

## Results

### mRNAs for *FDPS* and mevalonate pathway-related molecules were expressed in mouse taste buds

**Figure 1:**
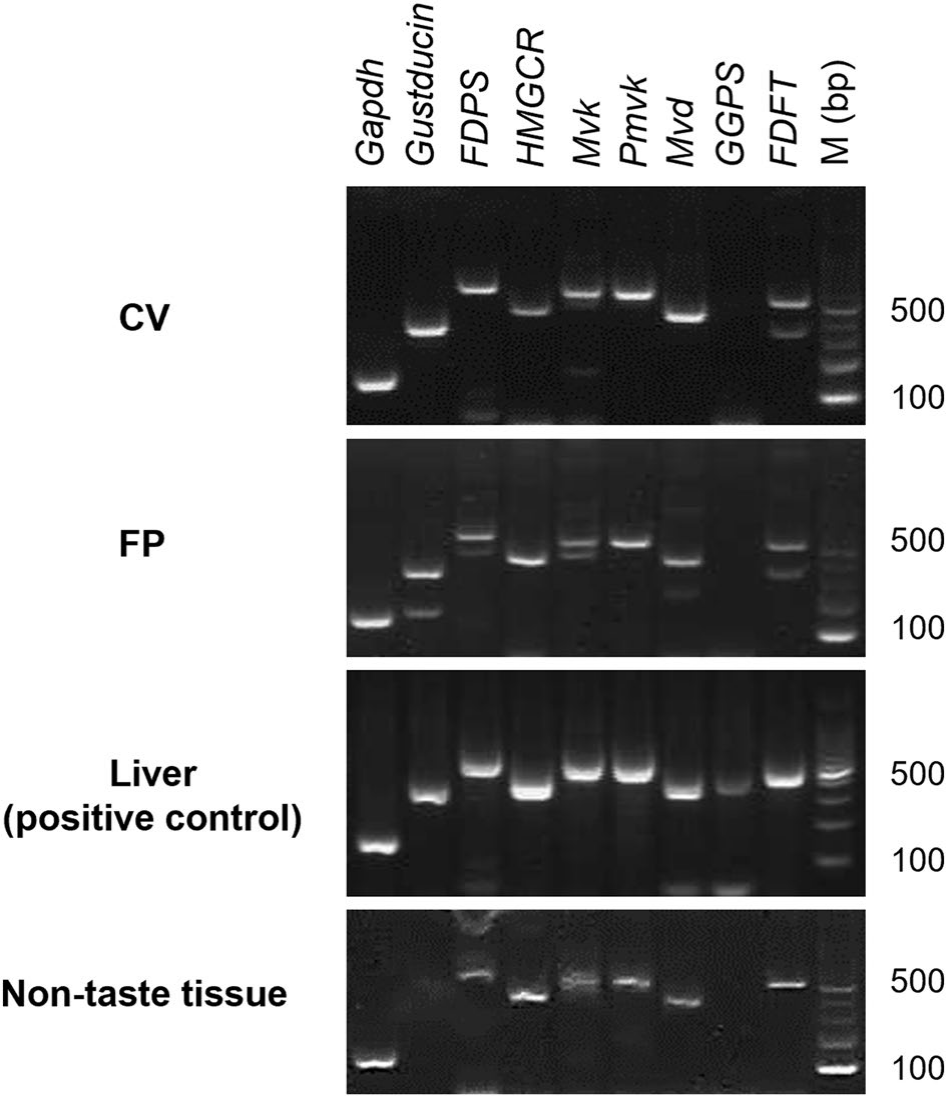
*Farnesyl diphosphate synthase* (*FDPS*) mRNA was expressed in mouse taste buds. Reverse transcription-polymerase chain reaction was used to amplify the mRNAs for mevalonate pathway component molecules from the circumvallate papillae (CV), fungiform papillae (FP), tongue non-taste epithelial tissue, and liver (positive control for mevalonate pathway components) of B6 mice. Mevalonate pathway components: *FDFT* squalene synthase, *GGPS* geranylgeranyl diphosphate synthase, *HMGCR* 3-hydroxy-3-methylglutaryl-CoA reductase, *Mvd* mevalonate pyrophosphate decarboxylase, *Mvk* mevalonate kinase, *Pmvk* phosphomevalonate kinase. *Gustducin* (a taste cell marker);* Gapdh* glyceraldehyde-3-phosphate dehydrogenase (housekeeping gene), *M* (*bp*) 100 bp marker ladder. Uncropped blots can be found in Supplementary Fig. S5.

We performed reverse transcription-polymerase chain reaction (RT-PCR) experiments to evaluate the mRNA expressions of *FDPS* and mevalonate pathway-related molecules in the circumvallate papillae (CV), fungiform papillae (FP), tongue non-taste epithelial tissue and liver (a positive control for mevalonate pathway-related molecules) of C57BL/6J (B6) mice (Fig. 1). A band of the correct size for *FDPS* mRNA (600 bp) was clearly detected in the taste tissues (CV and FP) and liver, slightly detected in non-taste tissues. In addition, other components of the mevalonate pathway, such as *HMGCR*, *Mvk*, *phosphomevalonate kinase* (*Pmvk*), m*evalonate pyrophosphate decarboxylase* (*Mvd*) and *FDFT*, were also detected. However, *geranylgeranyl diphosphate synthase* (*GGPS*) was not observed in mouse taste buds. RT-PCR products for *gustducin* (341 bp), a marker of type II taste cells, were found in both CV and FP taste buds but not in non-taste tissue, as expected. *Glyceraldehyde-3-phosphate dehydrogenase* (*Gapdh*) mRNA (150 bp) was detected in all tissues. All control experiments in which the reverse transcriptase enzyme was omitted yielded negative results.

### FDPS was expressed mainly in type III carbonic anhydrase-4 (CA4)-positive taste cells

**Figure 2:**
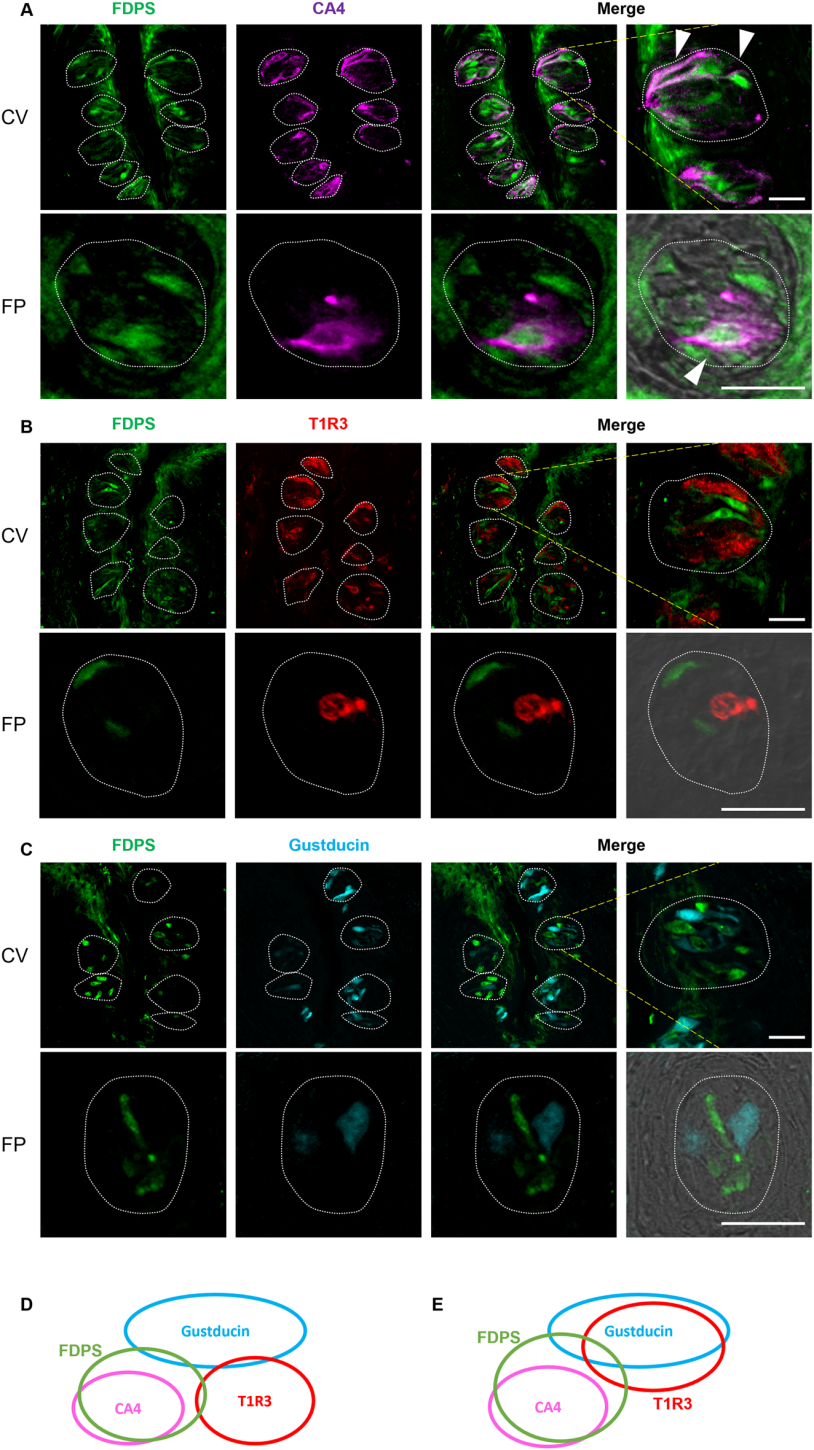
FDPS immunoreactivity (green) in the circumvallate papillae (CV) and the fungiform papillae (FP) of mice was observed mainly in taste bud type III cells. (**A**) Many FDPS-positive cells were also carbonic anhydrase-4 (CA4)-positive (magenta). Arrowheads denote FDPS + CA4 double-positive cells. (**B**,**C**) A few FDPS-positive cells also expressed taste receptor type 1 member 3 (T1R3, red) or gustducin (cyan). Dotted lines outline individual taste buds. Scale bars: 25 μm. (**D**,**E**) Summary of the pattern of co-expression between FDPS and taste cell markers in CV (**D**) and FP (**E**) taste cells. The area of each ellipse indicates the number of taste bud cells expressing FDPS, T1R3, gustducin or CA4 in the each taste papillae of the mouse. Regions of overlap indicate co-expression. This diagram is based on Table 1.

We performed double immunostaining experiments to investigate which taste bud cell types expressed FDPS after the antibody validation (Supplementary Fig. S1). A subset of FDPS-positive cells in the CV and FP expressed the type III taste cell marker, CA4^13^ (FDPS/CA4: 73.4% in CV; FDPS/CA4: 71.1% in FP; Fig. 2A), but a few FDPS-positive cells expressed the sweet/umami receptor component, T1R3 (FDPS/T1R3: 8.90% in CV; FDPS/T1R3: 14.9% in FP; Fig. 2B) or the bitter/sweet taste-related G protein, gustducin (FDPS/gustducin: 8.80% in CV; FDPS/gustducin: 11.5% in FP; Fig. 2C). A summary of the expression patterns of FDPS and taste cell markers is shown in Fig. 2D (CV) and Fig. 2E (FP). The inverse co-expression ratios (marker/FDPS) are shown in Table 1.

**Table 1:** Co-expression ratios for farnesyl diphosphate synthase (FDPS) and taste cell markers in mouse circumvallate and fungiform papillae.

Figure 2A–C	CV	FDPS/CA4	73.4%	(554/755, n = 213)	CA4/FDPS	66.7%	(554/831, n = 213)
		FDPS/T1R3	8.90%	(43/485, n = 147)	T1R3/FDPS	8.30%	(43/521, n = 147)
		FDPS/Gustducin	8.80%	(33/373, n = 101)	Gustducin/FDPS	8.10%	(33/409, n = 101)
	FP	FDPS/CA4	71.1%	(59/83, n = 60)	CA4/FDPS	57.8%	(59/102, n = 60)
		FDPS/T1R3	14.9%	(21/141, n = 86)	T1R3/FDPS	13.9%	(21/151, n = 86)
		FDPS/Gustducin	11.5%	(7/61, n = 56)	Gustducin/FDPS	6.3%	(7/112, n = 56)

The number of protein A + B positive cells/that of protein B positive cells, n = the number of taste buds analyzed.

**Figure 3:**
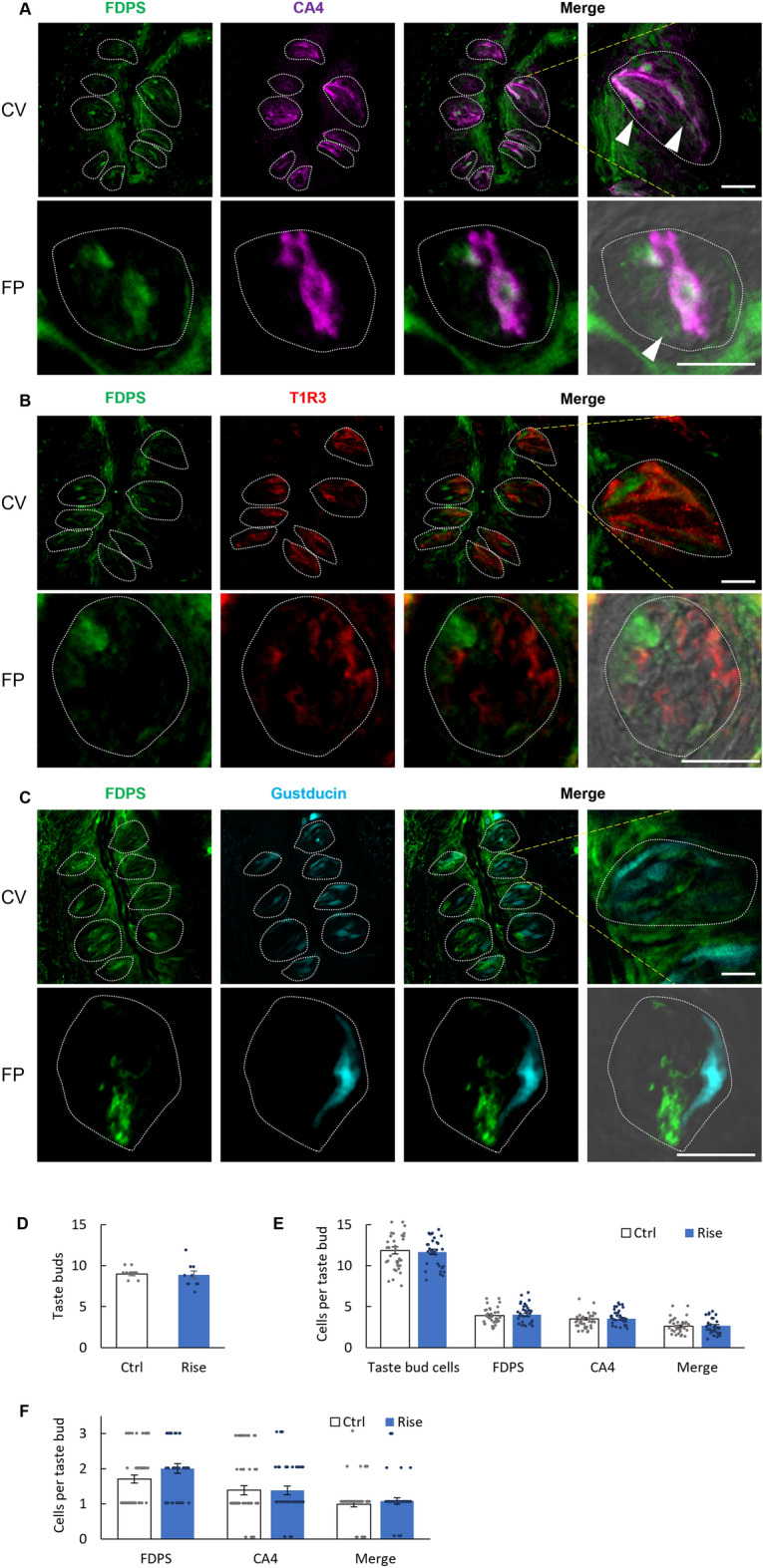
FDPS immunoreactivity (green) in the circumvallate papillae (CV) and the fungiform papillae (FP) of mice following the administration of risedronate (15 mg/kg body weight) for 28 days. (**A**) Many FDPS-positive cells were also carbonic anhydrase-4 (CA4)-positive (magenta). Arrowheads denote FDPS + CA4 double-positive cells. (**B**,**C**) A few FDPS-positive cells expressed taste receptor type 1 member 3 (T1R3, red) or gustducin (cyan). Dotted lines outline individual taste buds. Scale bars: 25 μm. (**D**) The number of taste buds after the administration of vehicle or risedronate for 28 days in the CV. The vehicle-treated group (Ctrl, white bar) and risedronate-treated group (Rise, blue bar) exhibited no significant differences in the number of taste buds. Data are presented as the mean ± SEM (n = 10 section slices from the middle part of 5 circumvallate papillae). *P* > 0.05 (Student's *t*-test; see Table 3). (**E**,**F**) The number of cells per taste bud after the administration of vehicle or risedronate for 28 days in the CV (**E**) and FP (**F**). The vehicle-treated group (Ctrl, white bars) and risedronate-treated group (Rise, blue bars) exhibited no significant differences in the number of taste bud cells in the CV, cells expressing FDPS, CA4 or both FDPS and CA4 (Merge). Data are presented as the mean ± SEM (n = 213–245 CV taste buds, n = 100–102 FP taste buds). *P* > 0.05 (Student's *t*-test; see Table 3).

Next, we compared the expression pattern and number of cells per taste bud between mice administered vehicle for 28 days and mice administered risedronate for 28 days. We confirmed that risedronate reached peripheral CV (Supplementary Fig. S2). The vehicle and risedronate groups exhibited no significant differences in the expression pattern (Fig. 3A–C, Table 2) or the number of taste buds (*P* > 0.05, *t*-test; Fig. 3D, Table 3), taste bud cells, FDPS-expressing, CA4-expressing or co-expressing cells (*P* > 0.05, *t*-test; Fig. 3E (CV) and F (FP), Table 3). Furthermore, comparing the number of FDPS-positive alone cells without co-expression of type II (gustducin) and type III (CA4) taste cell markers in the CV showed no significant difference between two groups (Supplementary Fig. S3).

**Table 2:** Co-expression ratios for farnesyl diphosphate synthase (FDPS) and taste cell markers in mouse circumvallate and fungiform papillae treated with risedronate three times a week for 28 days.

Figure 3A–C	CV	FDPS/CA4	75.8%	(639/843, n = 245)	CA4/FDPS	65.6%	(639/974, n = 245)
		FDPS/T1R3	7.90%	(27/343, n = 102)	T1R3/FDPS	7.50%	(27/360, n = 102)
		FDPS/Gustducin	10.4%	(45/433, n = 127)	Gustducin/FDPS	9.30%	(45/485, n = 127)
	FP	FDPS/CA4	78.3%	(54/69, n = 50)	CA4/FDPS	54.0%	(54/100, n = 50)
		FDPS/T1R3	19.1%	(26/136, n = 78)	T1R3/FDPS	16.0%	(26/163, n = 78)
		FDPS/Gustducin	18.9%	(14/74, n = 61)	Gustducin/FDPS	11.1%	(14/126, n = 61)

The number of protein A + B positive cells/that of protein B positive cells, n = the number of taste buds analyzed.

**Table 3:** Results of statistical analysis for the effect of injection of risedronate on the number of taste buds and the number of cells per taste bud (Fig. 3D–F).

Figure	Content	Analysis		P value
3D	Ctrl vs Rise	Student's *t*-test	Taste buds	0.845
3E	Ctrl vs Rise	Student's *t*-test	Taste bud cells	0.740
			FDPS	0.673
			CA4	0.999
			Merge	0.704
3F	Ctrl vs Rise	Student's *t*-test	FDPS	0.714
			CA4	0.515
			Merge	0.245

### Risedronate reduced the lick ratio for HCl but not for other basic taste solutions

**Figure 4:**
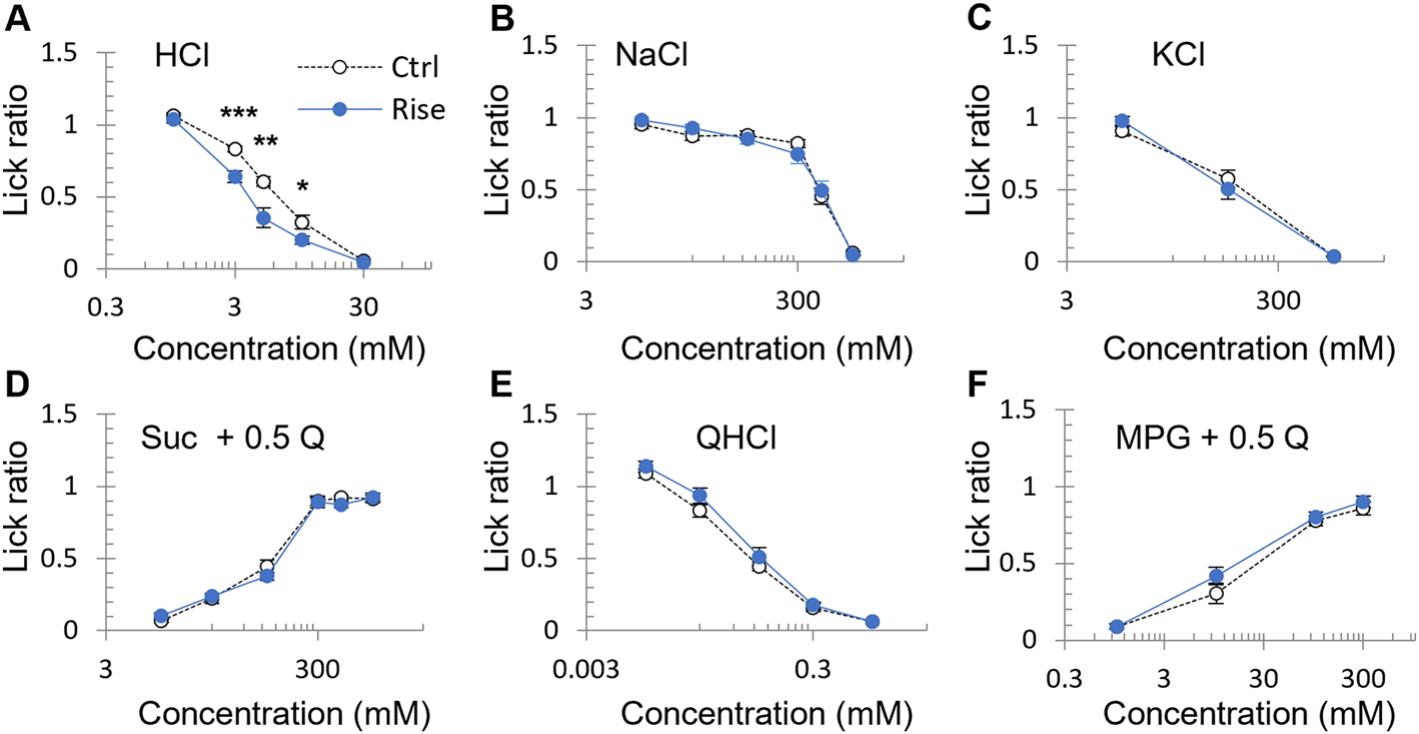
Risedronate enhanced the behavioral responses to HCl in mice. Lick ratios for varying concentrations of HCl (**A**), NaCl (**B**), KCl (**C**), sucrose combined with 0.5 mM quinine-HCl (Suc + 0.5 Q) (**D**), quinine-HCl (QHCl) (**E**) and monopotassium glutamate combined with 0.5 mM quinine-HCl (MPG + 0.5 Q) (**F**). The data were obtained after the intraperitoneal administration of vehicle (Ctrl, white symbols) or 15 mg/kg body weight risedronate (Rise, blue symbols) three times per week for 28 days. The lick ratio to distilled water is presented as the mean ± SEM (n = 9–21 mice). *P < 0.05, **P < 0.01, ***P < 0.001 (two-way ANOVA and post-hoc Student's *t*-test; see Table 4).

Next, we evaluated whether the administration of risedronate for 28 days affected the behavioral responses to various taste stimuli. There was no significant difference in the amount of water consumed during the 10-min test period between vehicle-treated and risedronate-treated mice (Supplementary Fig. S4). The lick ratio for HCl was significantly lower in risedronate-treated mice than in vehicle-treated mice (*P* < 0.05, ANOVA; Fig. 4A, Table 4). By contrast, the lick ratios for the other taste solutions (NaCl, KCl, sucrose + quinine-HCl [QHCl], QHCl, and monopotassium glutamate [MPG] + QHCl) were not affected by treatment with risedronate for 28 days (*P* > 0.05, ANOVA; Fig. 4B–F, Table 4).

**Table 4:** Results of statistical analysis for the effect of injection of risedronate on the lick ratio in mice (Fig. 4).

Figure	Content	Analysis			P value
4A	Injection (Ctrl vs Rise) × Concentration [HCl]	Two-way ANOVA	Injection (Ctrl vs Rise)	*F* (1,171) = 37.210	< 0.001
			Concentration	*F* (4,171) = 375.920	< 0.001
			Injection × Concentration	*F* (4,171) = 5.340	< 0.001
		Student's *t*-test	Ctrl vs Rise	1 mmol/L HCl	0.472
				3 mmol/L HCl	< 0.001
				5 mmol/L HCl	< 0.01
				10 mmol/L HCl	< 0.05
				30 mmol/L HCl	0.211
4B	Injection (Ctrl vs Rise) × Concentration [NaCl]	Two-way ANOVA	Injection (Ctrl vs Rise)	*F* (1,204) = 0.009	0.922
			Concentration	*F* (5,204) = 154.157	< 0.001
			Injection × Concentration	*F* (5,204) = 0.878	0.496
4C	Injection (Ctrl vs Rise) × Concentration [KCl]	Two-way ANOVA	Injection (Ctrl vs Rise)	*F* (1,66) = 0.001	0.997
			Concentration	*F* (2,66) = 227.363	< 0.001
			Injection × Concentration	*F* (2,66) = 1.364	0.262
4D	Injection (Ctrl vs Rise) × Concentration [Suc]	Two-way ANOVA	Injection (Ctrl vs Rise)	*F* (1,203) = 0.415	0.52
			Concentration	*F* (5,203) = 255.785	< 0.001
			Injection × Concentration	*F* (5,203) = 0.791	0.557
4E	Injection (Ctrl vs Rise) × Concentration [QHCl]	Two-way ANOVA	Injection (Ctrl vs Rise)	*F* (1.96) = 3.623	0.060
			Concentration	*F* (4.96) = 300.026	< 0.001
			Injection × Concentration	*F* (4.96) = 0.754	0.558
4F	Injection (Ctrl vs Rise) × Concentration [MPG]	Two-way ANOVA	Injection (Ctrl vs Rise)	*F* (1.86) = 2.438	0.122
			Concentration	*F* (3.86) = 163.876	< 0.001
			Injection × Concentration	*F* (3.86) = 0.686	0.563

### Risedronate did not affect the chorda tympani (CT) and the glossopharyngeal (GL) nerve responses to various taste solutions

**Figure 5:**
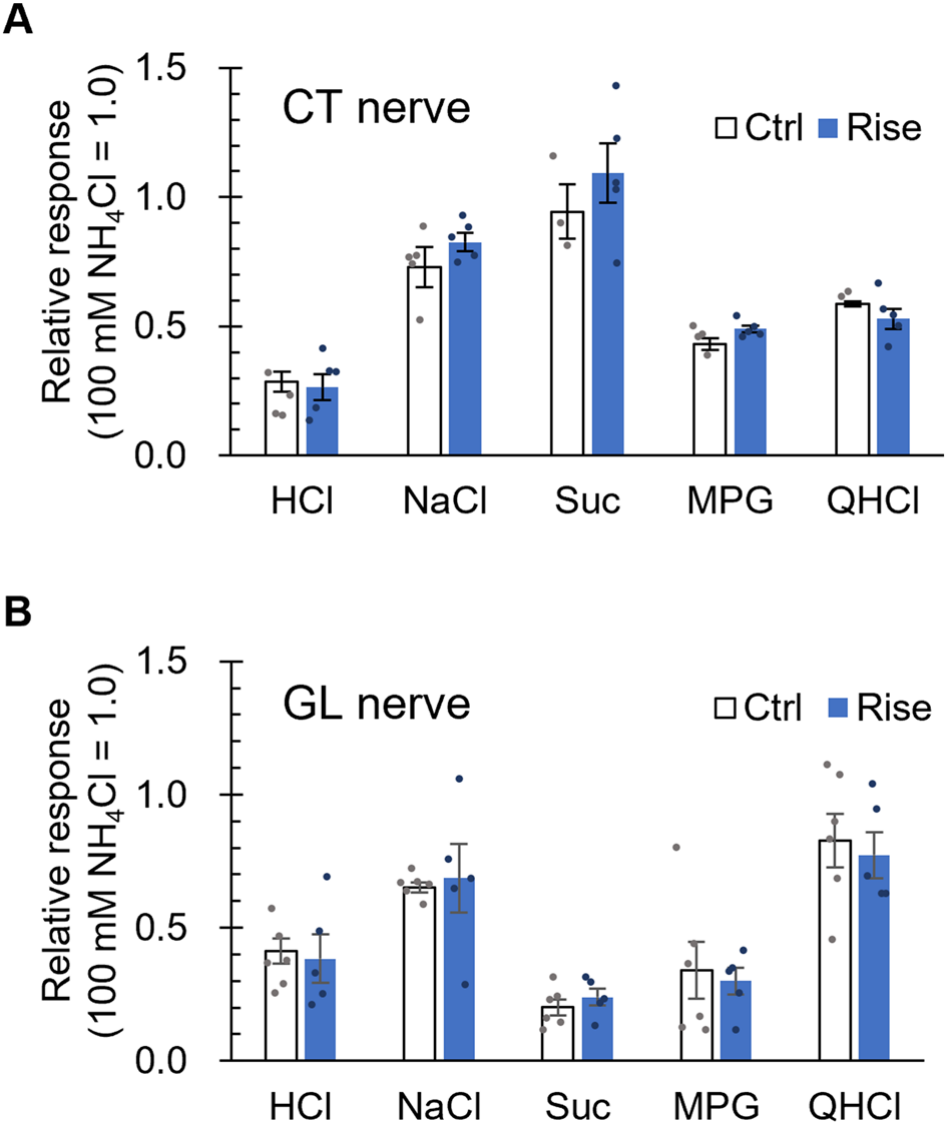
Risedronate did not affect the chorda tympani (CT) nerve and the glossopharyngeal (GL) nerve response to basic taste stimuli in mice. CT nerve (**A**) and GL nerve (**B**) responses to sour (3 mM HCl), salty (100 mM NaCl), sweet (300 mM sucrose [Suc]), umami (100 mM monopotassium glutamate [MPG]) and bitter (10 mM quinine-HCl [QHCl]) compounds were recorded after the administration of vehicle (Ctrl, white bars) or 15 mg/kg body weight risedronate (Rise, blue bars) three times per week for 28 days. Gustatory nerve responses were normalized to the response to 100 mM NH_4_Cl. Data are presented as the mean ± SEM (n = 3–5 mice). *P* > 0.05 (Student's *t*-test, see Table 5).

We further investigated whether treatment with risedronate for 28 days altered the CT and the GL nerve responses to various taste stimuli in mice. Unexpectedly, risedronate was without significant effect on both nerve responses to all taste solutions including HCl when compared with vehicle-treated mice (*P* > 0.05, *t*-test; Fig. 5A,B, Table 5).

**Table 5:** Results of statistical analysis for the effect of injection of risedronate on the CT and GL nerve response in mice (Fig. 5).

Figure	Content	Analysis		P value
5A	Ctrl vs Rise	Student's *t*-test	3 mmol/L HCl	0.419
			100 mmol/L NaCl	0.188
			300 mmol/L Suc	0.417
			100 mmol/L MPG	0.063
			10 mmol/L QHCl	0.416
5B	Ctrl vs Rise	Student's *t*-test	3 mmol/L HCl	0.386
			100 mmol/L NaCl	0.385
			300 mmol/L Suc	0.207
			100 mmol/L MPG	0.377
			10 mmol/L QHCl	0.351

### Risedronate decreased the *desmoglein-2* mRNA expression level in taste buds

**Figure 6:**
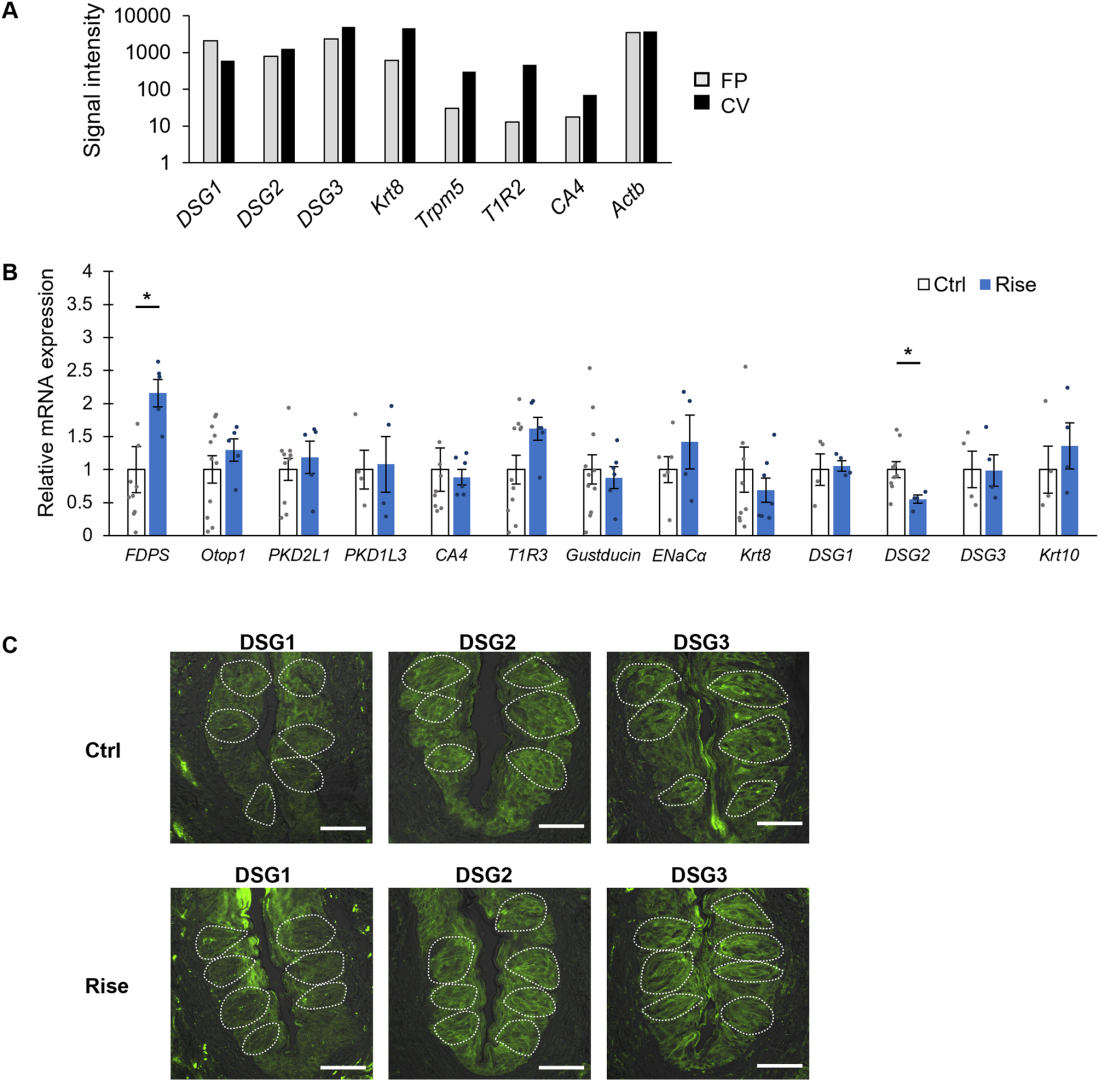
Risedronate significantly increased the expression level of *FDPS* mRNA and decreased the expression level of *desmoglein-2 (DSG2)* mRNA in mice. (**A**) DNA microarray analysis of the mRNA expressions of *desmoglein* (*DSG*) in non-treated mouse taste buds (fungiform and circumvallate papillae). Microarray analysis identified 3 subtypes of *DSG* (*DSG1, -2, -3*) that were positively expressed in both taste buds. Bar graph showing the microarray signal intensity for each gene in the fungiform papillae (FP, gray bars) and circumvallate papillae (CV, black bars). (**B**) Real-time PCR was used to determine the mRNA expressions of taste-related genes in the CV of mice administered vehicle (Ctrl, white bars) or 15 mg/kg body weight risedronate (Rise, blue bars) three times per week for 28 days. Data were obtained from at least three independent experiments per group, and each PCR assay was performed in duplicate. The quantitative PCR results were normalized using the ΔΔCt method with *glyceraldehyde-3-phosphate dehydrogenase* (*Gapdh*) as the reference and are shown as the fold-change in mRNA expression compared to the control. All data are presented as the mean ± SEM (n = 3–11 mice). *P < 0.05 (Student's *t*-test, see Table 6). (**C**) Expression of desmoglein-1 (DSG1), DSG2 and DSG3 in the CV of mice administered vehicle (Ctrl) or risedronate (Rise) for 28 days. The risedronate-treated mice exhibited no obvious morphological changes such as intercellular space enlargement or stratum corneum thinning in comparison to control mice. Immunostaining for DSG1/2/3 is shown in yellow-green. The immunostained images are overlaid with Nomarski images. Dotted lines outline individual taste buds. Scale bars: 50 μm. *Actb* actin beta, *CA4* carbonic anhydrase-4, *DSG1/2/3* desmoglein-1/2/3, *ENaC* epithelial sodium channel, *FDPS* farnesyl diphosphate synthase, *Krt8/10* keratin-8/10, *Otop1* otopetrin-1, *PKD* polycystic kidney disease, *T1R2/3* taste receptor type 1 member 2/3, *Trpm5* transient receptor potential cation channel subfamily M member 5.

Bisphosphonates have been shown to affect the expression of desmogleins (integral components of desmosomes that mediate intercellular adhesion) in the oral mucosa of bisphosphonate-treated patients and in primary human skin keratinocytes^8,14^. Therefore, we speculated that risedronate-induced taste disorders might involve a change in intercellular adhesion between taste cells that affects paracellular ion permeability^15,16^. Microarray analysis using isolated taste buds from non-treated mouse CV and FP showed that *desmoglein-1* (*DSG1*)*, DSG2* and *DSG3* mRNAs were positively expressed in an expression pattern similar to taste cell markers (*Krt8, Trpm5, T1R2* and *CA4*. The data of *T1R2* was from our previous study^17^) (Fig. 6A). To further explore this possibility, we examined the mRNA expression levels of *DSG1*, *DSG2*, *DSG3* and *keratin-10* (*Krt10*, a biomarker for keratinocyte terminal differentiation) in addition to those of *FDPS* and taste cell markers in taste bud cells.

The relative expression level of *FDPS* mRNA in taste bud cells was significantly higher in risedronate-treated mice than in vehicle-treated mice (*P* < 0.05, *t*-test; Fig. 6B, Table 6). On the other hand, the *DSG2* mRNA expression level was significantly lower in risedronate-treated mice than in vehicle-treated mice (*P* < 0.05, *t*-test; Fig. 6B, Table 6), whereas the expression levels of *DSG1*, *DSG3* and *Krt10* mRNA were comparable between groups (*P* > 0.05, *t*-test; Fig. 6B, Table 6). There were no significant differences between groups in the mRNA levels of type III cell markers (*Otop1*, *polycystic kidney disease-2L1 [PKD2L1]*, *PKD1L3* and *CA4*), type II cell markers (*T1R3* and *gustducin*), salty taste cell markers (*ENaCα*), and mature taste cell markers (*Krt8*) (*P* > 0.05, *t*-test; Fig. 6B, Table 6).

**Table 6:** Results of statistical analysis for the effect of injection of risedronate on the mRNA expression in mice (Fig. 6B).

Figure	Content	Analysis		P value
6B	Ctrl vs Rise	Student's *t*-test	*FDPS*	< 0.05
			*Otop1*	0.386
			*PKD2L1*	0.534
			*PKD1L3*	0.882
			*CA4*	0.785
			*T1R3*	0.073
			*Gust*	0.712
			*ENaCα*	0.330
			*Krt8*	0.484
			*DSG1*	0.833
			*DSG2*	< 0.05
			*DSG3*	0.974
			*Krt10*	0.499

Immunostaining experiments demonstrated the expressions of DSG1, DSG2 and DSG3 proteins in the CV taste cells and tongue non-taste epithelium of risedronate-treated mice after the antibody validation (Supplementary Fig. S1). However, risedronate-treated mice exhibited no obvious morphological changes, such as intercellular enlargement or thinning of the keratin layer, when compared with vehicle-treated control mice (Fig. 6C).

## Discussion

The aim of this study was to provide insights into the molecular mechanisms underlying bisphosphonate-induced taste disorders. It has been reported that oral or intravenous administration of etidronate (a bisphosphonate) can cause a transient taste disturbance in humans that is sometimes described as a “metallic taste”^3^. We speculated that bisphosphonate-induced taste disturbance might occur due to the inhibition of FDPS and downstream effects on the mevalonate pathway in taste bud cells. In this study, we revealed that FDPS is expressed in a subset of mouse taste bud cells. FDPS was expressed mainly in type III taste cells (CA4-positive cells mediating sour taste) and was slightly expressed in type II cells (T1R3-positive cells mediating sweet/umami taste or gustducin-positive cells mediating bitter taste), type I cells (without expression of both type II and type III taste markers), and non-taste tongue epithelial cells around taste buds, which suggests that FDPS in taste bud and tongue epithelial cells may be the target of bisphosphonates (Figs. 1, 2, 3).

The mevalonate pathway, also known as the HMG-CoA reductase pathway, produces two universal isoprenoid precursors, isopentenyl diphosphate and dimethylallyl diphosphate. These precursors are used to make isoprenoids, a large and highly diverse class of at least 20,000 biomolecules such as cholesterol, bile acids and steroid hormones. The mevalonate pathway involves HMGCR, Mvk, Pmvk, Mvd, GGPS and FDFT in addition to FDPS, as mentioned above^18,19^. The present study found that all the mevalonate pathway genes except that for *GGPS* were expressed in both CV and FP taste tissues and tongue epithelium of the mouse, which indicates that the mevalonate pathway mediates cholesterol synthesis through *FDFT* in taste bud and tongue epithelial cells (Figs. 1, 2, 3). Previous publications describing whole transcriptome profiling of taste bud cells have reported that the expression levels of *HMGCR*, *FDPS* and *FDFT* were higher in type III cells than in type II (T1R3-positive) cells, which supports our results^12^. Type III cells are known to be not only sour responsive but also neuronal cells with visible synaptic structures^20^. Cholesterol is an essential structural component of cell membranes and myelin, and cholesterol is necessary for synapse and dendrite formation, axonal guidance and cell function. Depletion of neuronal cholesterol impairs synaptic vesicle exocytosis, neuronal activity and neurotransmission and leads to the degeneration of dendritic spines and synapses^21^. Moreover, statins, which are widely prescribed cholesterol-lowering drugs, are potentially teratogenic. For example, *HMGCR* deficiency causes preimplantation lethality in mice^22^, *Mvk* deficiency causes early embryonic lethality in mice^23^, and the absence of *FDFT* is fatal at E9.5–12.5 d.p.c.^24^. It has also been reported that the rapid reduction of cholesterol levels in Madin-Darby canine kidney cells by methyl-β-cyclodextrin alters paracellular permeability, likely due to disruption of the tight junction network^25^. These results suggest that cholesterol synthesized through the mevalonate pathway may help to sustain the normal functioning of type III taste cells by maintaining cell viability, synaptic formation and the passage of ions and other solutes through the paracellular route.

The mevalonate pathway is involved not only in cholesterol production but also in the synthesis of isoprenoid lipids such as farnesyl pyrophosphate (FPP) and geranylgeranyl diphosphate (GGPP), which are substrates for posttranslational lipid modification and prenylation of proteins. Prenylation is required for various proteins to function properly, because lipid isoprenyl groups anchor proteins to the cell membrane and are thought to be involved in protein–protein interactions^26^. Osteoclasts are the main target of bisphosphonates in the treatment of osteoporosis, and the prenylation of small GTPases (such as Ras, Rho and Rac) in osteoclasts contributes to the regulation of various cellular processes such as maintenance of cell morphology, integrin signaling, membrane ruffling, endosome transport and apoptosis^27–30^. Bisphosphonates almost completely inhibit the prenylation of these small GTPases in osteoclasts, which induces apoptotic cell death and thereby slows bone loss^31,32^. In our RT-PCR analysis, *GGPS* mRNA was barely detected in taste tissues, suggesting that the mevalonate pathway in taste bud cells may not be involved in the production of GGPP and protein prenylation (Fig. 1). Notably, the administration of risedronate for 28 days did not alter the number of taste buds, taste bud cells, FDPS-positive and CA4-positive taste cells per taste bud or the magnitudes of the taste nerve responses (CT and GL) to basic tastants (Figs. 3D,E, 5). These results suggest that bisphosphonate-induced taste disturbance might not be due to apoptotic changes in taste bud cells secondary to inhibition of protein prenylation, as is the case for osteoclasts.

Bisphosphonates have been shown to affect the adhesion of various types of cells including epithelial cells^8^, endothelial cells^33^ and cancer cells^34^. It has also been reported that bisphosphonates are associated with esophagitis and oral ulceration^8,26,35,36^. Experiments using in vitro models of the oral mucosa have shown that pamidronate and zoledronate reduce the viability, inhibit the proliferation and enhance the apoptosis of oral keratinocytes and fibroblasts. Furthermore, bisphosphonates also reduced epithelial thickness and prevented epithelial formation in three-dimensional tissue-engineered models of the oral mucosa^37–39^. Donetti et al. reported that alendronate decreased the proliferation of keratinocytes, reduced the expressions of *DSG1* and *Krt10*, and impaired the structure of desmosomes in human oral epithelium without causing widening of the intercellular spaces or changes in the expression patterns of the tight junction markers, E-cadherin and occludin^8^. Treatment of primary human skin keratinocytes with zaragozic acid, a FDFT inhibitor that blocks the conversion of FPP to squalene, was found to reduce epidermal differentiation and the levels of cell adhesion and junctional molecules such as Krt10, DSG1 and vinculin^14^. Our microarray study showed that *DSG1*, *DSG2* and *DSG3* mRNAs were expressed in both the CV and FP of mice (Fig. 6A). Therefore, we speculated that bisphosphonate-induced taste disorders might involve changes in intercellular adhesion between taste cells that affect paracellular ion permeability in the taste buds^15,16^. Evaluation of the expressions of *DSG*s in taste buds demonstrated that mice administered risedronate for 28 days exhibited a significantly higher expression of *FDPS* mRNA and a significantly lower expression of *DSG2* mRNA in the taste buds (Fig. 6B). Our data acquired in a mouse model appear to be consistent with those obtained from the healthy oral mucosa of alendronate-treated patients^8^. Previous research has shown that an antibody directed against DSG2 and siRNA-mediated *DSG2* depletion caused reductions in cell adhesion and epithelial barrier function in monolayers of Caco-2 cells (a human colorectal adenocarcinoma cell line) and that DSG2 expression was strongly reduced in the mucosa of patients with Crohn’s disease^40,41^. The above findings imply that DSG2 is required for epithelial barrier function and may contribute to barrier dysfunction in Crohn's disease. The results of the present study suggest that risedronate may selectively inhibit the FDPS function in the taste bud and tongue epithelial cells and thereby reduce the production of DSG2. The enhanced expression of *FDPS* mRNA in risedronate-treated mice may be a genetic compensatory mechanism in response to the decreased levels of proteins regulated by FDPS. These results suggest that the reduction of DSG2 in the taste bud and tongue epithelial cells may affect the epithelial barrier function, which causes abnormal paracellular permeability to H^+^ and/or Cl^−^, resulting in an enhancement of the behavioral response to HCl and weak acids such as citric acid and acetic acid via somatosensory nerves in and/or around the taste buds (Fig. 4). The taste alteration (e.g. metallic taste) observed in patients treated with bisphosphonates may be related to the effects of H^+^ and/or Cl^−^ on somatosensation.

In conclusion, the findings of this study indicate that the mevalonate pathway, which mediates cholesterol synthesis, is functional in mouse taste bud cells. Furthermore, risedronate may interact directly with FDPS expressed in taste bud and tongue epithelial cells to inhibit *DSG2* expression, which may alter paracellular permeability to H^+^ and/or Cl^−^ in the taste buds and tongue epithelium, resulting in enhancement of an aversive response to HCl mediated via somatosensory nerves. These findings provide new insights into the mechanisms by which bisphosphonates cause changes in taste perception in humans.

## Materials and methods

### Animals

Mouse husbandry and all mouse experiments were carried out in accordance with the ethical guidelines of Kyushu University. All experimental protocols and procedures were approved by the Committee for Laboratory Animal Care and Use at Kyushu University (approval no. A20-003-0, 16 December 2019) in accordance with the ARRIVE guidelines. B6 mice were purchased from Charles River Laboratories Japan (Yokohama, Japan). Transgenic α-gustducin-green fluorescent protein (GFP) mice expressing GFP under the control of the α-gustducin promoter were a gift from Dr. Robert F. Margolskee, Monell Chemical Senses Center (Philadelphia, PA, USA)^42^. All mice were housed under specific pathogen free conditions on a 12/12-h light/dark cycle at 23 °C and had ad libitum access to water and food pellets (CE-2, CLEA Japan, Tokyo, Japan). Both male and female mice aged 8–12 weeks were used for the experiments. Risedronate (15 mg/kg body weight) or vehicle (control) were given by intraperitoneal injection three times per week for 28 days^43^. Risedronate is one of the most widely used bisphosphonates and an inhibitor of FDPS^44^. Risedronate is often used long-term for the treatment of osteoporosis and Paget's disease^45–47^. It has been reported that taste bud cells turn over in a 10–14-day cycle^48,49^. Undifferentiated cells, which reside in the basal portion of the taste buds, migrate to the central portion of the taste bud to become mature functional cells with taste receptors, and eventually these cells undergo cell death^50,51^. Thus, a taste bud contains a mixture of cells at different stages of maturation. Given the turn-over cycle of 10–14 days, it would be expected that the taste bud cells would be fully affected by risedronate after 28 days of treatment.

### Drugs and taste compounds

Risedronate (LKT Laboratories, St Paul, MN, USA) was dissolved in vehicle (0.9% NaCl). The drug concentrations were adjusted to provide the appropriate dose in a total volume of 1.0 mL. The dose of risedronate administered was based on that reported previously^43^.

The following taste solutions were used in this study: NaCl, HCl, KCl, sucrose (with or without QHCl), QHCl, NH_4_Cl (all purchased from Fujifilm Wako Pure Chemical Corporation, Osaka, Japan) and MPG (Sigma-Aldrich, St Louis, MO, USA). All taste solutions were dissolved in distilled water (DW)^52^.

### Reverse transcription-polymerase chain reaction (RT-PCR)

RT-PCR was performed as previously described^52–54^. The tongues were dissected from B6 mice (n = 3) and divided into anterior and posterior segments, which were injected with 0.3 mL dispase α and 0.1 mL dispase α (0.1 U/mL; Sigma-Aldrich, St Louis, MO, USA), respectively, and incubated for 10 min at 37 °C. The epithelia of the anterior and posterior parts of the tongue were peeled away, injected with elastase (0.28 U/mL; Elastin Products Company, Owensville, MO, USA) and incubated for 7 min at 37 °C. Then, the surrounding tissue was removed, and the taste buds in the FP (50 taste buds per mouse) were collected with a glass pipette and placed in Tyrode solution (in mM: NaCl 140, KCl 5, CaCl_2_ 1, MgCl_2_ 1, NaHCO_3_ 5, glucose 10, sodium pyruvate 10, HEPES 10; pH 7.4 adjusted with NaOH). Tissue containing the CV was dissected from the surrounding tissue, and the Von Ebner's glands were removed under a microscope.

The RNAs of mouse taste tissue were extracted using the FastGene RNA Premium Kit (Nippon Genetics, Tokyo, Japan), and RNA purity was assessed through determination of the A260/A280 ratio using a NanoDrop ND-1000 spectrophotometer (Thermo Fisher Scientific, Waltham, MA, USA). SuperScript VILO Master Mix (Thermo Fisher Scientific, Waltham, MA, USA) was used for cDNA synthesis. The PCR conditions were as follows: 15 min at 95 °C (1 cycle); 30 s at 94 °C, 30 s at 57 °C, and 60 s at 72 °C (35 cycles); and 5 min at 72 °C (1 cycle). Each 20-μL reaction mixture contained 0.5 U Taq DNA polymerase (TaKaRa Ex Taq HS, Takara, Shiga, Japan) and 2 μL 10 × PCR buffer containing 20 mM Mg^2+^, 0.6 mM of each primer pair, 0.2 mM of each dNTP, and 0.4 μL cDNA solution. The amplified products were visualized under UV illumination using gel electrophoresis (2% agarose with GelRed Nucleic Acid Gel Stain, Biotium, Fremont, CA, USA). Purified RNA samples were processed with or without reverse transcriptase in parallel to control for signals from genomic DNA. All primer pairs were selected such that each primer was from a different exon. The primers used for each gene are listed in Table 7.

**Table 7:** Primers used for RT-PCR.

Gene	Forward primer	Reverse primer	Product size	Accession no.
*Gapdh*	TGTGTCCGTCGTGGATCTGA	TTGCTGTTGAAGTCGCAGGAG	150	NM_001289726
*Gustducin*	TGCTTTGAAGGAGTGACGTG	GTAGCGCAGGTCATGTGAGA	341	NM_001081143
*FDPS*	GGCTTGGTTTCCTACAACGA	GCTCCAGCAGGTTCAGGTAG	600	NM_001253751.1
*HMGCR*	CGAAGGACGAGGAAAGACTG	GCCATCACAGTGCCACATAC	447	NM_001360165.1
*Mvk*	CCTCTCACCCACCTGTTTGT	GAACTTGGTCAGCCTGCTTC	580	NM_001306205.1
*Pmvk*	GCGAGCACCTACAAGGAGAC	CCTCTCCAGGGAAAACACAA	577	NM_001310640.1
*Mvd*	GCTCAGGTCTATGGGGTTGA	CCTGGAGGTGTCATTGAGGT	432	NM_138656.2
*GGPS*	TGAGGGTCCTGATTGGCTAC	TAGCCAAGTGGGGTGTTAGG	438	NM_001331119.1
*FDFT*	CTTGGTTCCTCTGCCTCTTG	GCACTGCCTGCTTTCCTTAC	527	NM_001360211.1

### Immunohistochemistry

The dissected tongues of B6 mice (n = 17 control mice, n = 18 treated mice) and α-gustducin-GFP mice (n = 7 control mice, n = 8 treated mice) were fixed in 4% paraformaldehyde in phosphate-buffered saline for 45 min at 4 °C and then dehydrated with a graded series of sucrose solutions (10% for 1 h, 20% for 1 h, and 30% for 3 h at 4 °C). A frozen block of tongue tissue was embedded in optimal-cutting-temperature compound (Sakura Finetechnical, Tokyo, Japan) and sectioned into 10-μm-thick slices, which were mounted on silane-coated glass slides and air-dried. The sections were washed with Tris–NaCl–Tween (TNT) buffer three times (5 min for each wash) and incubated for 1 h in Blocking One solution (Nacalai Tesque, Kyoto, Japan). Then, the sections were incubated with the primary antibodies overnight at 4 °C. After washing with TNT buffer, the slides were incubated in secondary antibodies for 2 h and then washed again. The immunofluorescence of labeled cells and GFP fluorescence were observed with a laser-scanning microscope (FV-1000, Olympus, Tokyo, Japan), and images were captured using Fluoview software (Olympus, Tokyo, Japan). The number of taste buds, taste bud cells, FDPS-expressing, CA4-expressing, T1R3-expressing, and gustducin-expressing cells were determined by counting positively stained cells in each taste bud in horizontal sections of the CV and FP. Artificial signals were excluded using Image-Pro Plus v4.0 (Media Cybernetics, Rockville, MD, USA). Cells were considered positive if their signal density was greater than the mean + 2 standard deviations of the signal density of taste cells in the negative control (primary antibody omitted). The same cells found in consecutive sections were counted only once. The primary antibodies used in this study were rabbit anti-FDPS (1:100; Thermo Fisher Scientific, Waltham, MA, USA), goat anti-CA4 (1:100; R&D Systems, Minneapolis, MN, USA), goat anti-T1R3 (1:100; Santa Cruz Biotechnology, Dallas, TX, USA), rabbit anti-DSG1 (1:100; Abcam, Cambridge, UK), rabbit anti-DSG2 (1:100; Abcam, Cambridge, UK) and rabbit anti-DSG3 (1:100; Bioss, Woburn, MA, USA). The secondary antibodies used were Alexa Fluor 488 or 568 donkey anti-rabbit IgG (1:300; Invitrogen, Waltham, MA, USA) for immunostaining of FDPS, DSG1, DSG2 and DSG3, and Alexa Fluor 568 donkey anti-goat IgG (1:300; Invitrogen, Waltham, MA, USA) for immunostaining of T1R3 and CA4. Negative controls were obtained by replacing the primary antibodies with the antibody diluent in Blocking One solution to assess any background signal (Supplementary Fig. S1).

### Behavioral test

We used short-term (10 s) tests to study the effects of risedronate on behavioral responses. Details of the procedures used for this test are described in our previous publications^52,54,55^. Each B6 mouse (n = 9–21 control mice, n = 9–19 treated mice) was deprived of water for 23 h, placed in a test box on day 1 of training and given free access to DW during a 1 h session. The licks were detected by a lick meter equipped with a laser beam lick sensor (Yutaka Electronics, Nagoya, Japan) and recorded on a strip chart recorder. During the training sessions on days 2–5, the mice were trained to drink DW at scheduled intervals, which consisted of 10 s periods of DW presentation alternated with 20 s intervals when DW was not available. From day 6, the number of licks for each test stimulus and for DW were counted during the first 10 s after the mouse's first lick.

Measurements of the number of licks were made for mice who had received intraperitoneal injections of either vehicle (physiological saline) three times per week for 28 days or risedronate (15 mg/kg body weight) dissolved in vehicle three times per week for 28 days. On each test day, the first test stimulus given to the mouse was DW, and then the following solutions were tested in a randomized order: 1–30 mM HCl, 10–1000 mM NaCl, 10–1000 mM KCl, 10–1000 mM sucrose with 0.5 mM QHCl, 0.01–1 mM QHCl, and 1–300 mM MPG with 0.5 mM QHCl. 0.5 mM QHCl was added to each sucrose test solution and MPG test solution to enable concentration-dependent changes in the lick rates for sweeteners and umami to be determined more clearly^52–54,56^. The mean value of the tastant/DW lick ratio for each test stimulus was calculated for each mouse.

### Gustatory nerve recording

Gustatory nerve responses to the lingual application of taste solutions were recorded from the CT and GL nerves as described previously^52,54,55^. All procedures were performed under pentobarbital anesthesia (50–60 mg/kg body weight). Each B6 mouse (n = 3–6 control mice, n = 5 treated mice) was fixed in the supine position with its head in a holder to allow dissection of the CT or the GL nerve. The right CT nerve was freed from the surrounding tissues after removal of the pterygoid muscle and cut at the point of its entry to the bulla. The right GL nerve was exposed by removal of the digastricus muscle and posterior horn of the hyoid bone. The GL nerve was then dissected free from underlying tissues and cut near its entrance to the posterior lacerated foramen. The entire nerve was placed on an Ag/AgCl electrode, and indifferent electrodes were placed in nearby tissue. Neural activity was fed into an amplifier (K-1; Iyodenshikagaku, Nagoya, Japan) and monitored on an oscilloscope and audio monitor. Whole nerve responses were integrated with a time constant of 1.0 s and recorded on a computer using a PowerLab/sp4 system (AD Instruments, Bella Vista, NSW, Australia). The anterior half of the tongue was enclosed in a silicone rubber flow chamber to allow stimulation of the FP. For taste stimulation of the CV and the foliate papillae, an incision was made on each side of the animal's face from the corner of the mouth to just above the angle of the jaw, then the papillae exposed and their trenches opened by slight tension applied through a small suture sewn in the tip of the tongue. Taste solutions (100 mM NH_4_Cl, 3 mM HCl, 100 mM NaCl, 300 mM sucrose, 100 mM MPG or 10 mM QHCl) were delivered to each part of the tongue by gravity flow for 30 s (CT) or 60 s (GL). The tongue was washed with DW for ~ 60 s between successive stimulations. Only stably recorded data were used in the analysis. In the analysis of whole-nerve responses, integrated whole-nerve response magnitudes were measured 5, 10, 15, 20 and 25 s (for the CT) and 5, 10, 20, 30 and 40 s (for the GL) after stimulus onset. There values were averaged, and normalized to the response to 100 mM NH_4_Cl to account for inter-animal variations in the absolute responses.

### DNA microarray analysis

Taste buds were collected from non-treated B6 mice. Total RNA extraction was performed as described above for RT-PCR. The GeneChip Mouse Genome 430 2.0 Array (Affymetrix, Santa Clara, CA, USA) was used for microarray analysis^17^. RNA quality control, total RNA labeling, microarray hybridization and scanning were performed in accordance with the Affymetrix GeneChip Expression Analysis Technical Manual (Thermo Fisher Scientific, Waltham, MA, USA).

### Quantitative polymerase chain reaction (qPCR)

The taste buds isolated from each CV of each B6 mouse (n = 4–11 control mice, n = 3–7 treated mice) were pooled. Total RNA extraction was performed as described above for RT-PCR. The RNA concentration was measured using a NanoDrop ND-1000 spectrophotometer (Thermo Fisher Scientific, Waltham, MA, USA). qPCR was performed using Fast SYBR Green Master Mix and an ABI StepOnePlus system (Applied Biosystems, MA, USA). The conditions used for PCR were: 50 °C for 2 min and 95 °C for 2 min (1 cycle); 95 °C for 3 s and 60 °C for 30 s (40 cycles); and 95 °C for 15 s, 60 °C for 1 min, and 95 °C for 15 s (1 cycle for melting curve analysis). StepOne 2.3 (Applied Biosystems, Waltham, MA, USA) was used for data analysis. The presence of a single amplicon was confirmed by melting curve analysis and agarose gel electrophoresis. Data were obtained from at least three independent experiments, and all reactions were performed in duplicate. The qPCR data were normalized using the ΔΔCt method with *Gapdh* in each sample as the reference^57^. ΔΔCt values were calculated by subtracting the average ΔCt value for the control sample from the respective ΔCt values for the vehicle-treated and risedronate-treated samples. Fold change was calculated as 2^(−ΔΔCT)^. All primer pairs were selected so that each primer was from a different exon. The qPCR primers used for each gene are presented in Table 8.

**Table 8:** Primers used for qPCR.

Gene	Forward primer	Reverse primer	Product size	Accession no
*FDPS*	TCCTTCTGCCCATAATTCTCC	GGTGGTTCAGTGTCTGCTACG	72	NM_001253751.1
*Otop1*	GCCAACAGCGTCCTGAATGA	TCCAAAACGATGGTGATGTTGC	95	NM_172709.3
*PKD2L1*	CAGATGCGACAGGGACTGG	GGGGCTGTGTCCAACAGAC	84	NM_181422
*PKD1L3*	TCCCACATCAGCTCAGAAGAAC	ATGGGGTGCTGTTCCATGTC	141	NM_001039700.3
*CA4*	TACTGAAGACTCAGGCTGGTG	GGTCATAGCCGACGAGGATG	179	NM_007607.2
*T1R3*	CAAGGCCTGCAGTGCACAA	AGGCCTTAGGTGGGCATAATAGGA	92	NM_031872
*Gustducin*	AGGGCATCTGAATACCAGCTCAA	CTGATCTCTGGCCACCTACATCAA	196	NM_001081143
*ENaCα*	GCTTCATCTTTACCTGTCGTTTC	CCAGAGATTGGAGTTGTTCTTGT	122	NM_001406028.1
*Krt8*	TGAACAACAAGTTCGCCTCCTT	GCTCCTCGACGTCTTCTGCT	110	NM_031170
*DSG1*	TCGTGGATGTAACAGAAGCCA	TGGCGTTATGATCTCTTACCTGG	133	NM_010079.2
*DSG2*	GGGCCACTCACCATGTAAGG	TGTAGGAGGCCGCTTTCTCT	162	NM_007883.3
*DSG3*	TCAGCCCGAAGACAAGGAAATC	TCTACCATGGTCCCACCAGC	120	NM_030596.4
*Krt10*	GACAACTGACAATGCCAACG	GCCAGCTCTTCGTTCAGACT	204	NM_010660.2
*Gapdh*	TGTGTCCGTCGTGGATCTGA	TTGCTGTTGAAGTCGCAGGAG	150	NM_001289726

### Statistical analysis

Measurement data are shown as the mean ± standard error of the mean (SEM). Data for the behavioral test were compared using factorial two-way ANOVA and post-hoc Student's *t*-tests. Data for the other experiments were compared using Student's *t*-tests. The level of significance was taken as *P* < 0.05. Statistical comparisons were made using SPSS Statistics 19 (IBM, Armonk, NY, USA).

## Supplementary Information

Supplementary Information 1.

Supplementary Information 2.

## Data Availability

All data generated or analyzed during this study are included in this published article (and its supplementary information 1.docx and 2.xlsx).

## Abbreviations

B6: C57BL/6J
CA4: Carbonic anhydrase-4
CT: Chorda tympani
CV: Circumvallate papillae
DSG1/2/3: Desmoglein-1/2/3
DW: Distilled water
ENaC: Epithelial sodium channel
FDFT: Farnesyl-diphosphate farnesyl transferase (squalene synthase)
FDPS: Farnesyl diphosphate synthase
FP: Fungiform papillae
FPP: Farnesyl pyrophosphate
Gapdh: Glyceraldehyde-3-phosphate dehydrogenase
GGPP: Geranylgeranyl diphosphate
GGPS: Geranylgeranyl diphosphate synthase
GL: Glossopharyngeal
GFP: Green fluorescent protein
HMGCR: 3-Hydroxy-3-methylglutaryl-CoA reductase
Krt8/10: Keratin-8/10
MPG: Monopotassium glutamate
Mvd: Mevalonate pyrophosphate decarboxylase
Mvk: Mevalonate kinase
Otop1: Otopetrin-1
PKD: Polycystic kidney disease
Pmvk: Phosphomevalonate kinase
QHCl: Quinine-HCl
qPCR: Quantitative polymerase chain reaction
RT-PCR: Reverse transcription-polymerase chain reaction
SEM: Standard error of the mean
T1R1/2/3: Taste receptor type 1 member 1/2/3
TNT: Tris–NaCl–tween
