# Supplementary material for: Bisphosphonate affects the behavioral responses to HCl by disrupting farnesyl diphosphate synthase in mouse taste bud and tongue epithelial cells
Source: Sci Rep. 2022 Dec 8;12:21246. doi: 10.1038/s41598-022-25755-5 (PMC9732047; doi:10.1038/s41598-022-25755-5)
Supplement: Supplementary file 1 — Supplementary Information 1. [file 41598_2022_25755_MOESM1_ESM.docx]

**Supplementary information**

Bisphosphonate affects the behavioral responses to HCl by disrupting farnesyl diphosphate synthase in mouse taste bud and tongue epithelial cells.

Asami Oike^a,b^, Shusuke Iwata^a,c^, Ayaka Hirayama^a^, Yurika Ono^a^, Yuki Nagasato^a^, Yuko Kawabata^d^, Shingo Takai^a^, Keisuke Sanematsu^a,c,e^, Naohisa Wada^b^, Noriatsu Shigemura^a,c,*^.

^a^ Section of Oral Neuroscience, Graduate School of Dental Science, Kyushu University, Fukuoka, Japan

^b^ Section of Interdisciplinary Dentistry, Graduate School of Dental Science, Kyushu University, Fukuoka, Japan

^c^ Research and Development Center for Five-Sense Devices, Kyushu University, Fukuoka, Japan

^d^ Department of Cell Biology, Aging Science, and Pharmacology, Division of Oral Biological Sciences, Faculty of Dental Science, Kyushu University, Fukuoka, Japan

^e^ Oral Health/Brain Health/Total Health Research Center, Faculty of Dental Science, Kyushu University, Fukuoka, Japan

* shigemura@dent.kyushu-u.ac.jp

**Supplementary Figure. 1**

Positive and negative controls for validation of antibody for FDPS, DSG1, DSG2 and DSG3. FDPS- and DSG-signals were detected in the liver ^1^ and tongue epithelium ^2–4^ (as positive control) in B6 mice, respectively. Negative control experiments in which the 1^st^ antibody for FDPS, DSG1, DSG2 and DSG3 were omitted yielded negative results in circumvallate papillae (CV), liver and tongue epithelium.


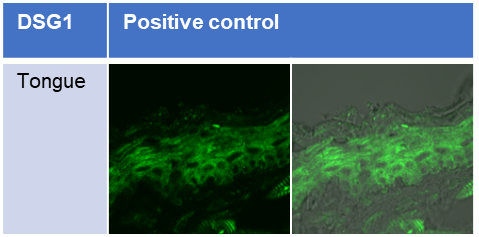

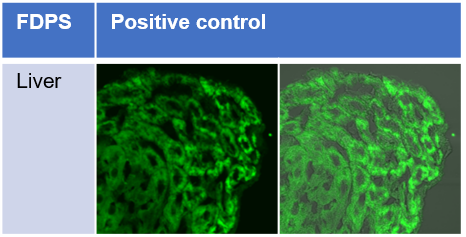

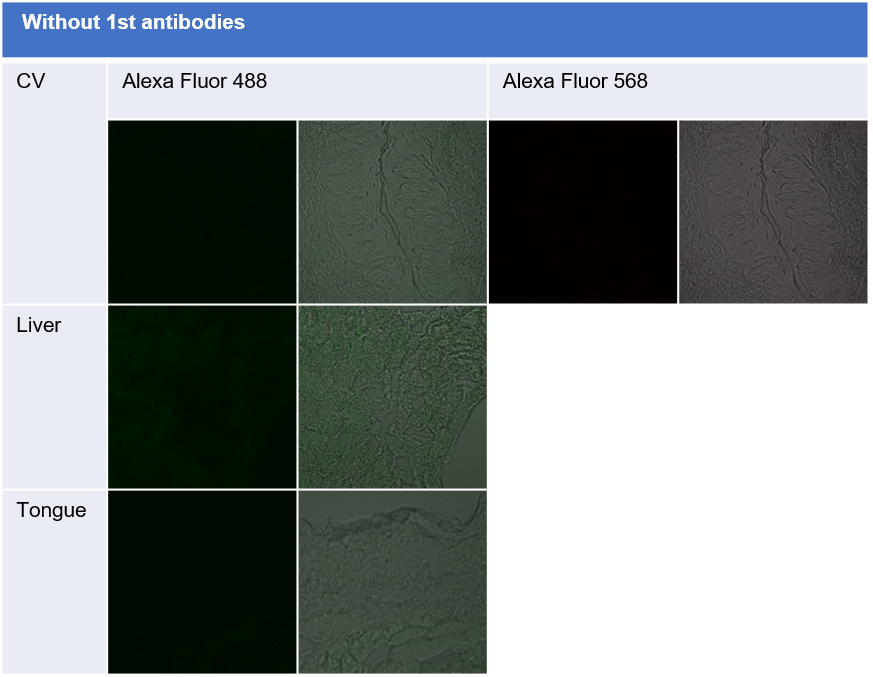

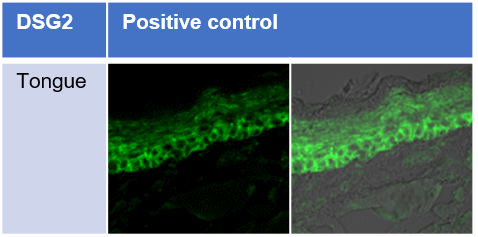

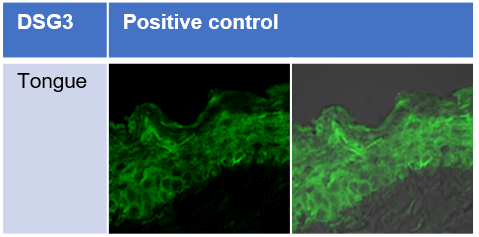


**Supplementary Figure. 2**

Risedronate reached circumvallate papillae (CV) in vivo. Alexa Fluor 647-labeled risedronate (AF647-RIS), a fluorescent risedronate analog, was used. The tongue and liver tissues of 3 month old female B6 mice intraperitoneally injected with vehicle or AF647-RIS (0.9 mg/kg; Invitrogen, Paisley, UK) were excised 30 min after injection. The dose of AF647-RIS administered was based on that reported previously^1^. The peeled tongue epithelium was pinned out in a Sylgard-coated culture dish. Following fixation, the CV and liver tissues were scanned on a laser-scanning microscope (FV-1000, Olympus, Tokyo, Japan) using the 680 nm laser to detect AF647-RIS. As a result, AF647 signals were detected in CV and liver (positive control ^1^) treated with AF647-RIS, not in those treated with vehicle. (A) Fluorescence images of control tissue injected with vehicle. (B) Fluorescence images of tissue 30 min after injection of AF647-RIS. (C) Magnified images of CV in (B). Scale bars: 100 μm.

**
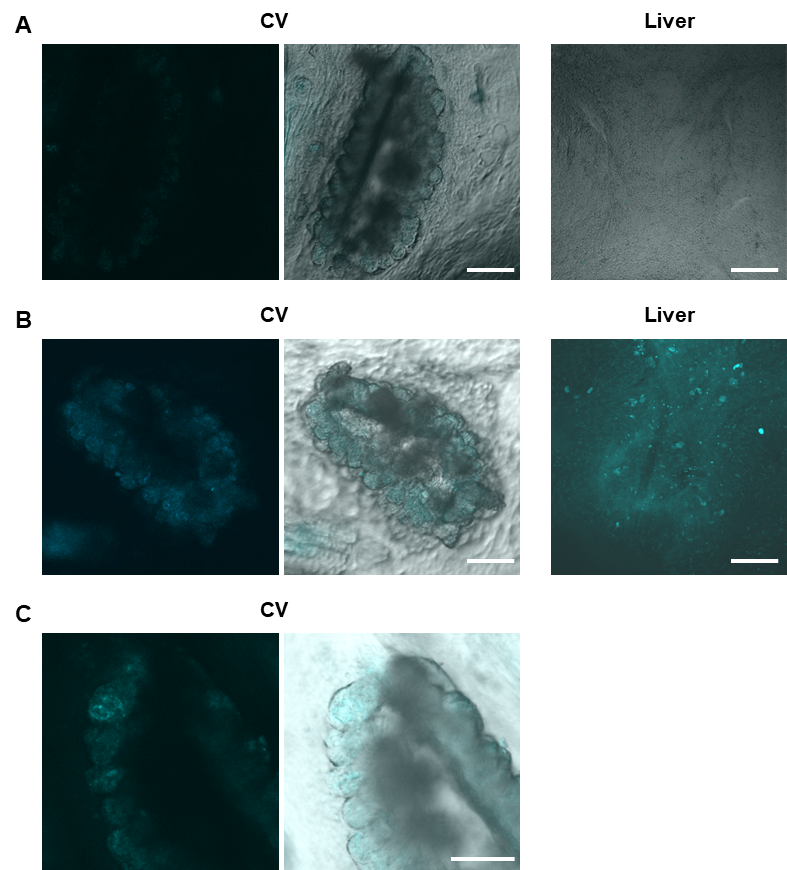
**

**Supplementary Figure. 3**

FDPS immunoreactivity (green) without co-expression of carbonic anhydrase-4 (CA4) and gustducin in mouse circumvallate papillae (CV). (A) Most FDPS-positive cells were also CA4-positive (magenta). A few FDPS-positive cells expressed gustducin (cyan). Dotted lines outline individual taste buds. Scale bars: 50 μm. (B) High magnification image of merge. Arrowheads denote FDPS-positive alone cells. Scale bars: 25 μm. (C) The number of FDPS-positive alone cells per taste bud after the administration of vehicle or risedronate for 28 days in the CV. The vehicle-treated group (Ctrl, white bars) and risedronate-treated group (Rise, blue bars) exhibited no significant differences in the number of FDPS-positive alone cells. Data are presented as the mean ± SEM (n = 101–127 taste buds). *P* > 0.05 (Student's *t*-test).

**
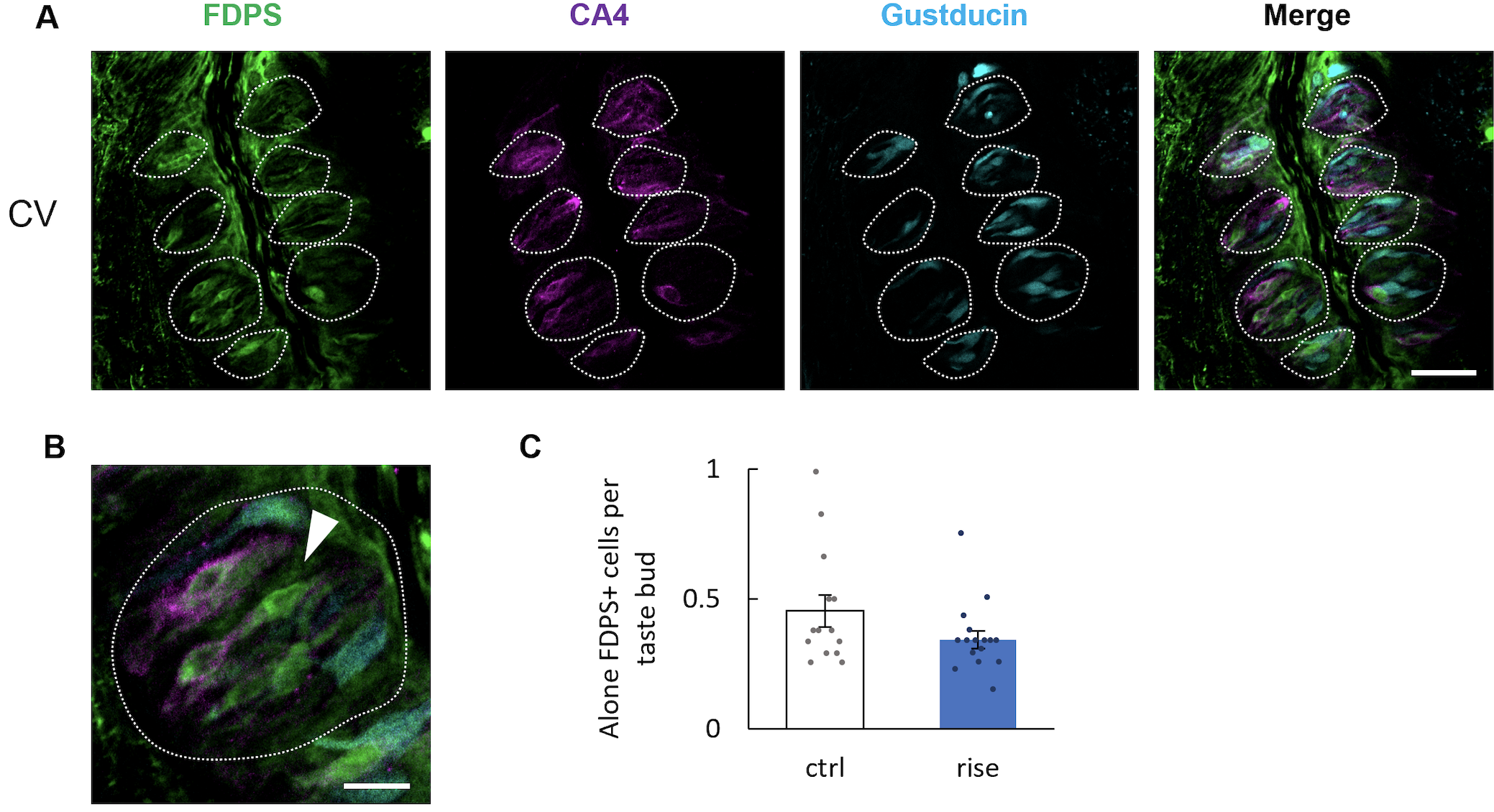
**

**Supplementary Figure. 4**

The effect of risedronate on water drinking behavior in mice. The data were obtained after the intraperitoneal administration of vehicle (Ctrl, white bar) or 15 mg/kg body weight risedronate (Rise, blue bar) three times per week for 28 days. Each B6 mouse was deprived of water for 23 h prior to testing. Access to food was never restricted. There was no significant difference in the amount of water consumed during the 10-min test period between vehicle- and risedronate-treated mice. Data are presented as the mean ± SEM (n = 7–9 mice). *P* > 0.05 (Student's *t*-test).


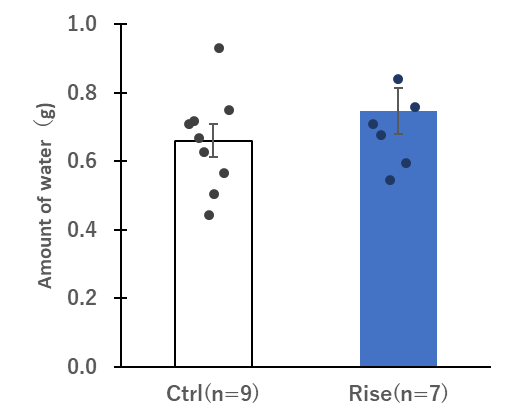


**Supplementary Figure. 5**

Full length gel images of RT-PCR experiments (Fig 1. Farnesyl diphosphate synthase [FDPS] mRNA was expressed in mouse taste buds). Areas enclosed by red dot line were used in Fig 1.


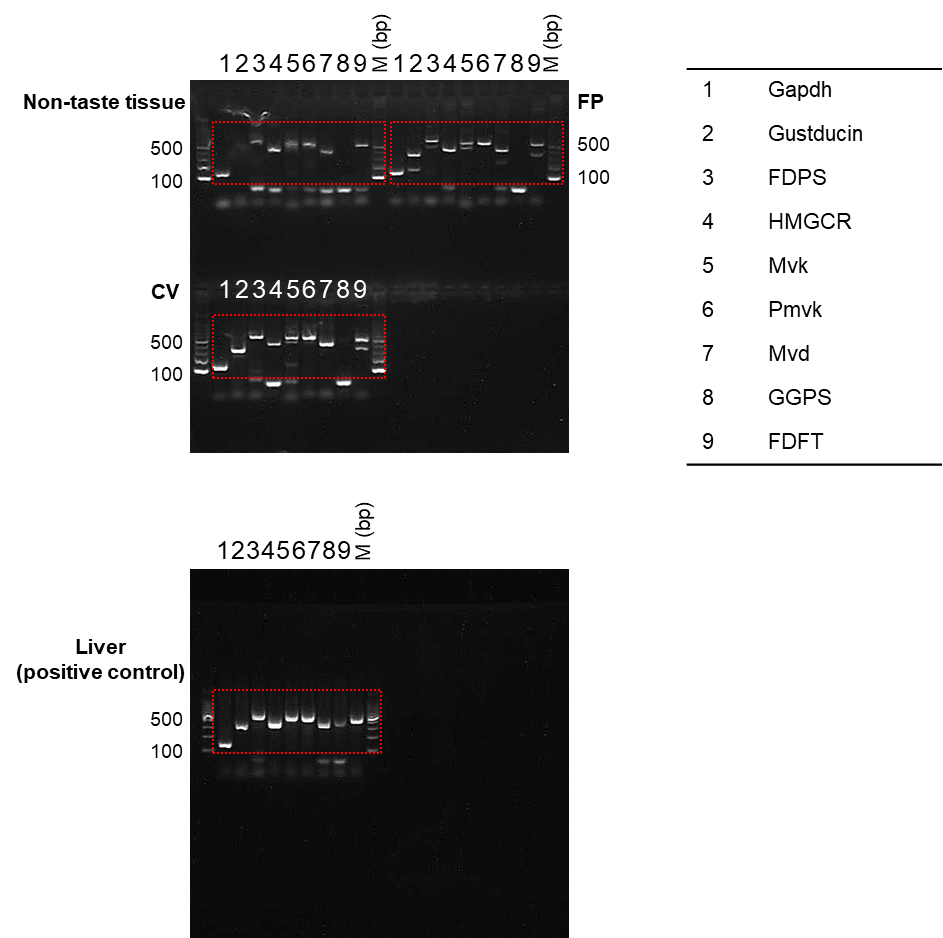


**Reference**

1. Roelofs, A. J. *et al.* Fluorescent risedronate analogues reveal bisphosphonate uptake by bone marrow monocytes and localization around osteocytes in vivo. *J Bone Miner Res* **25**, 606–616 (2010).

2. Marzouki, S. *et al.* Implicating bites from a leishmaniasis sand fly vector in the loss of tolerance in pemphigus. *JCI Insight* **5**, (2020).

3. Brennan, D. *et al.* Suprabasal Dsg2 expression in transgenic mouse skin confers a hyperproliferative and apoptosis-resistant phenotype to keratinocytes. *J Cell Sci* **120**, 758–771 (2007).

4. Koch, P. J. *et al*. Targeted disruption of the pemphigus vulgaris antigen (desmoglein 3) gene in mice causes loss of keratinocyte cell adhesion with a phenotype similar to pemphigus vulgaris. *Journal of Cell Biology* **137**, 1091–1102 (1997).
